# Circumferential Decompression Technique of Posterior Endoscopic Cervical Foraminotomy

**DOI:** 10.1155/2022/5873333

**Published:** 2022-01-24

**Authors:** Guo-Li Hou, Chien-Min Chen, Kuo-Tai Chen, San-En Xu, Lin Tao, Ling-Tong Kong, Guo-Zhong Lai, Lei Shi, Lei Chu, Ying-Dong Chen

**Affiliations:** ^1^Department of Orthopedics, Dehong Hospital of Traditional Chinese Medicine, Yunnan, China; ^2^Department of Neurosurgery, Changhua Christian Hospital, Changhua City, Taiwan; ^3^Department of Neurosurgery, Chang Gung Memorial Hospital, Chiayi, Taiwan; ^4^Department of Orthopedics, Second Affiliated Hospital of Chongqing Medical University, Chongqing, China

## Abstract

**Objective:**

Cervical osseous foraminal stenosis (COFS) results from the uncinate process and facet hyperostosis. Currently, the optimal surgical technique for the treatment of COFS remains controversial.

**Materials and Methods:**

Patients with COFS presenting radiculopathy underwent posterior endoscopic cervical foraminotomy by the circumferential decompression technique. The neck disability index (NDI), the visual analogue scale (VAS), and the modified MacNab criteria were used to evaluate the outcomes. In addition, the range of motion (ROM) and the slippage distance between the operated vertebrae in flexion-extension position were measured to evaluate the stability of the cervical spine.

**Results:**

There were 24 consecutive patients in the study. The mean follow-up period was 16.2 months (range: 12-26 months). The NDI and VAS scores for arm/neck pain improved significantly from preoperatively to the last follow-up. The satisfaction rate by modified MacNab criteria was 91.7% on the third postoperative day and 100% on the day of final follow-up. There were no significant differences in intervertebral ROM or slippage distance between the last follow-up and preoperatively (*P* = 0.968, *P* = 0.394). Arm pain occurred in one patient, and sustained fingers numbness in two patients, but these symptoms resolved at the last follow-up.

**Conclusions:**

Posterior endoscopic cervical foraminotomy by the circumferential decompression technique is a safe and effective treatment for COFS. Moreover, it preserves the stability and physiological mobility of the cervical spine.

## 1. Introduction

Cervical osseous foraminal stenosis (COFS), defined by osseous foraminal narrowing of the cervical spine, is commonly caused by the uncinate process and facet hyperostosis or lateral soft disc herniation [1–3]. The progressive narrowing of the intervertebral foramina caused by these pathological changes may lead to nerve root impingement, inflammation, and cervical radiculopathy.

Anterior cervical discectomy and fusion (ACDF) is considered the gold standard surgical treatment for the treatment of COFS. Decompression is performed from the front, and intervertebral bone graft fusion can increase the height of the intervertebral foramina to a certain extent. However, there are still different opinions regarding whether the anterior osteophyte from the uncinate process should be removed [4–6].

Some researchers [7–10] have reported that minimally invasive surgery via the anterior approach can remove the osteophyte of the uncinate process and achieve anterior nerve decompression. However, the anterior approach is not widely used in clinical practice, and spine surgeons prefer the posterior approach. In recent years, posterior endoscopic cervical foraminotomy or discectomy has become a gradually popular alternative for treating cervical intervertebral foramen stenosis [11–17]. However, most studies have focused on treating soft disc herniation and hypertrophic facet joints. Few studies have demonstrated how to resolve nerve compression due to osseous foraminal stenosis with the posterior approach.

In this study, we proposed a novel circumferential decompression technique based on the “keyhole” approach to treat COFS and evaluated the clinical feasibility, safety, and effectiveness of this technique.

## 2. Materials and Methods

### 2.1. General Information

This study was a retrospective study. Twenty-four patients with COFS were included in this study from May 2017 to May 2019. The institutional review board approved the current study (No. 2017-1). Written informed consents were obtained from all the patients. All the patients had symptoms and signs of nerve root entrapment in the corresponding segments and failed to improve with at least three-month conservative treatment. 11 male and 13 female patients with an average age of 50.8 years (range, 38-68). The mean duration of symptoms was 7.1 months (ranged: 3-24 months). The level of COFS was C4/5 in 6 cases, C5/6 in 13 cases, and C6/7 in 5 cases. None of the cases had a surgical history before this study. The demographic characteristics of the cases are shown in Table 1.

### 2.2. Inclusion and Exclusion Criteria

**Figure 1:**
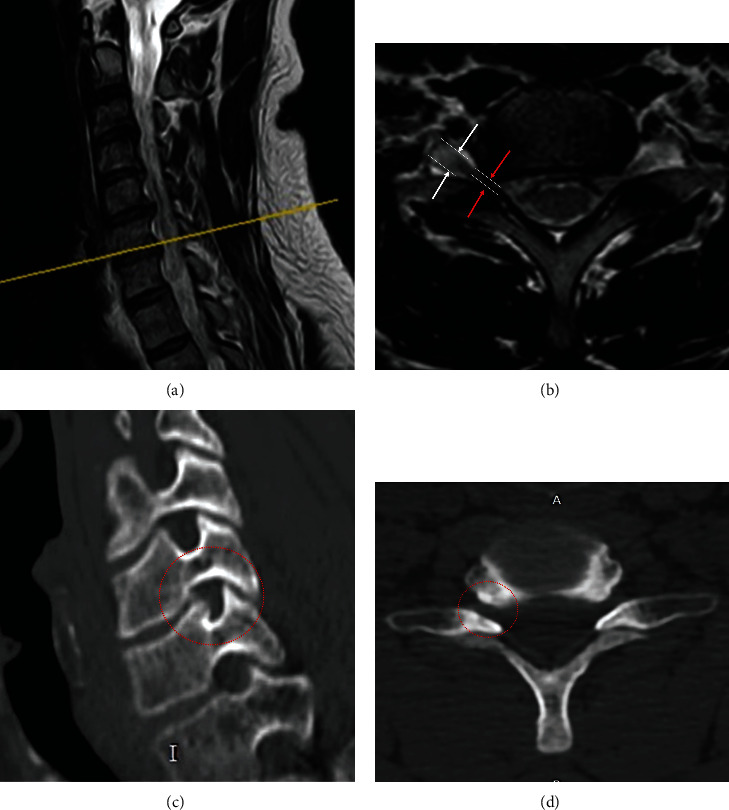
Cervical intervertebral foraminal stenosis at C6-7 level. The illustration of MRI in (a) sagittal and (b) transverse view. CT images in (c) sagittal and transverse view (red arrow indicates the width of the stenotic intervertebral foramina, and white arrow indicates the width of the nerve root at the outer foramina).

The inclusion criteria were as follows: [1] typical clinical symptoms and signs consistent with single segment nerve root injury; [2] sustained or repeated symptoms after more than six weeks of conservative treatment [15, 16]; and [3] COFS confirmed by X-ray, CT, and MRI (Figure 1).

The exclusion criteria were as follows: [1] cervical spondylotic myelopathy; [2] severe cervical spinal canal stenosis; [3] ossification of the posterior longitudinal ligament; [4] definitive segmental instability; [4] suspected infection or tumor in the cervical spine; and [5] congenital cervical deformities.

### 2.3. Operative Technique

The same surgeon performed all operations. Under local anesthesia combined with intravenous analgesics, the patients were placed prone with the head fixed in a radiolucent holder. The patient remained awake throughout the surgical procedure, and a neurophysiological monitor was unnecessary. Then, the targeted vertebral level was confirmed by C-arm fluoroscopy. The vertical anchoring technique was used to locate the entry point [18].

A stab incision was made at the targeted level, and a working cannula was docked by C-arm fluoroscopic guidance. The endoscope of 7 mm outer diameter with a 4.3 mm working channel (SPINENDOS GmbH, Munich, Germany) was applied in the procedure. At first, a radiofrequency bipolar tip (Vantage Technology Co., Ltd., Taoyuan) and a grasper were used to dissect the soft tissues overlying the lamina and facet joint to expose the “V-point” (the overlapping intersection of the upper and lower lamina). Generally, a 3 mm diameter burr (Guizhou Zirui Technology, Guizhou, China) was used to open the V-point. A keyhole approximately 6 mm in diameter was drilled under endoscopic visualization. The keyhole was mainly to unroof the intervertebral foramen from the posterior approach. The nerve root was exposed, and the soft disc herniation could be removed from the axilla of the nerve root. However, circumferential decompression should be evaluated first with preoperative imaging. Parts of the hyperplastic facets should be properly removed, but the area of facetectomy should be limited to 50% to avoid cervical spine instability [19]. After the dorsal foraminotomy, hemostasis and dissection in the spinal canal were essential to exposing the nerve root. Radiculopathy was usually associated with disc herniation, and it was commonly located in the axilla of the nerve root. The inferior part of the nerve root was decompressed by removing the herniated disc. Subsequently, the osteophyte on the ventral side of the nerve root was exposed.

**Figure 2:**
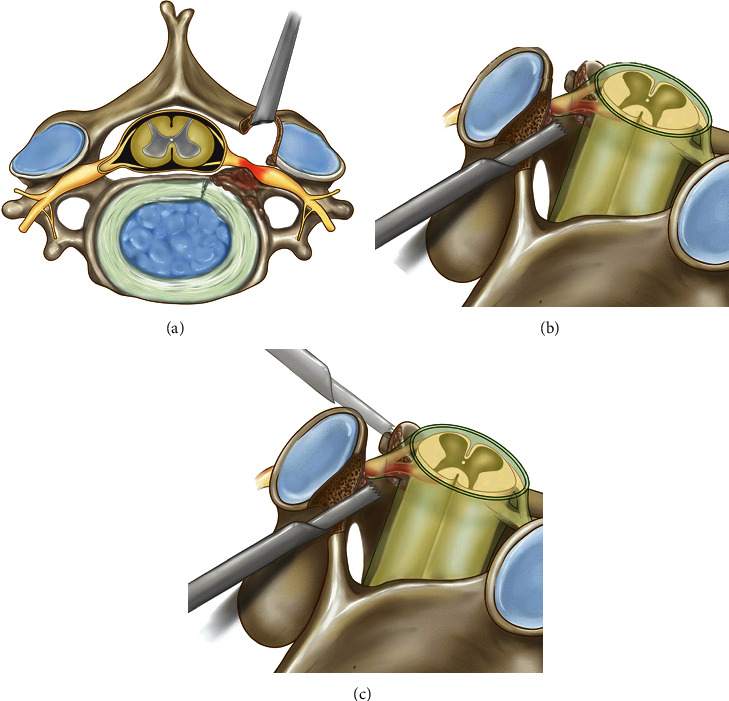
Diagrammatic drawing of circumferential decompression. (a) Posterior indirect decompression. (b) Decompression from the axilla of the nerve root. (c) Decompression from the shoulder of the nerve root.

However, COFS usually occurs in the middle part of the foramen, and it is difficult to achieve satisfactory clinical results if only posterior indirect decompression is performed (Figure 2(a)). Moreover, further expanding the intervertebral foramen might compromise spinal instability because more articular processes need to be removed. We evaluated preoperative CT images and identified features of uncovertebral joint hyperplasia that caused nerve root impairment in the intervertebral foramen (Figures 2(b) and 2(c)). The planned trajectory depended on the location of the spiculate on the uncinate process.

**Figure 3:**
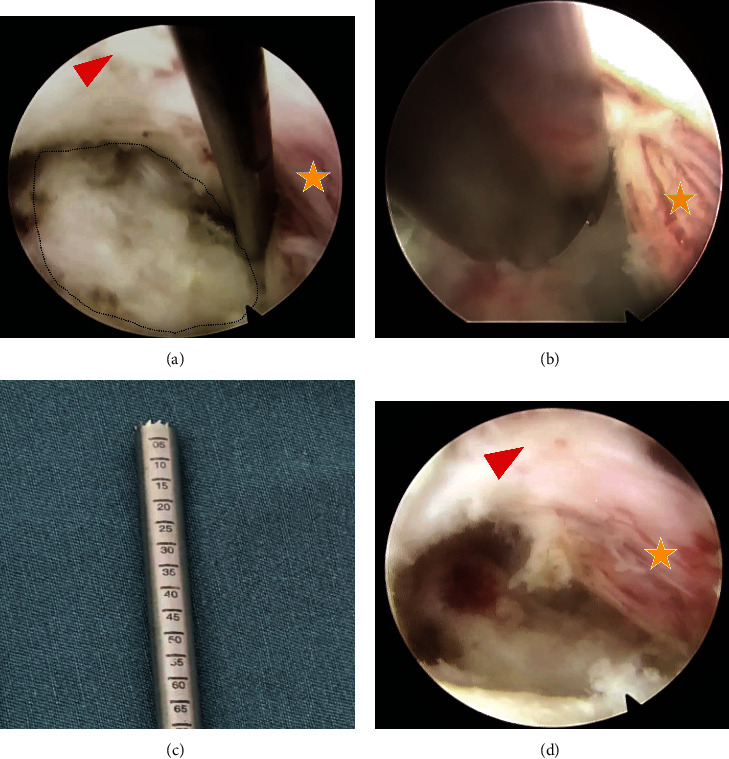
Images comparison of preoperation and after circumferential decompression under full-endoscope. (a) Before osteophyte removal. (b) Using endoscopic trephine. (c) The trephine with scale ruler can help to determine the depth while removing osteophytes. (d) After osteophyte removal (the red triangle represents the spinal cord, the arrow indicates the head direction, and the star represents the nerve root).

When hyperplasia occurred at the base of the uncinate process, it was usually located in the axilla region of the nerve root. Therefore, the starting point for establishing the bone tunnel was often lower than the V-point, the tunnel was created through the lower lamina by the cranial and lateral inclination to the axillary of the nerve root, and part of the pedicle was removed. Then, a nerve probe was used to detect the shape and scope of the osteophyte from the axilla of the nerve root (Figure 3(a)). When nerve root compression occurred at the tip of the uncinate process, a bony tunnel in the upper lateral mass was created to remove the tip of the uncinate process from the shoulder of the nerve root (Figure 2(c)).

Subsequently, a 3 mm endoscopic trephine was used to perpendicularly reach the surface of the target osteophyte (Figure 3(b)). Before the trephine was rotated, the endoscopic angle of view, the dura and nerve root were visualized, and entrapment under the trephine was avoided. The depth and extent of osteophyte removal were determined according to the preoperative CT images. Commonly, there was a scale ruler on the surface of the trephine (Figure 3(c)), and we usually checked the depth of the trephine tip by the ruler. Meanwhile, we could recheck the intraoperative fluoroscopy to assure that the tip of the trephine passes through the posterior vertebral line. After the osteophytes were removed, the mobility of the nerve root was rechecked using the probe. Consequently, nerve root decompression was performed (Figure 3(d)).

### 2.4. Postoperative Treatment

The patients were ambulatory 24 hours later after surgery. All patients were encouraged to wear a neck collar for six weeks. Because this technique involved more bony structure removal than the conventional technique, partial pedicle or lateral mass removal led to fracture risk. Therefore, it was necessary to limit the movement of extension and flexion. Clinical follow-ups were carried out at one week, six weeks, three months, six months, and 12 months postoperatively.

### 2.5. Efficacy Evaluation and Observation Indicators

**Figure 4:**
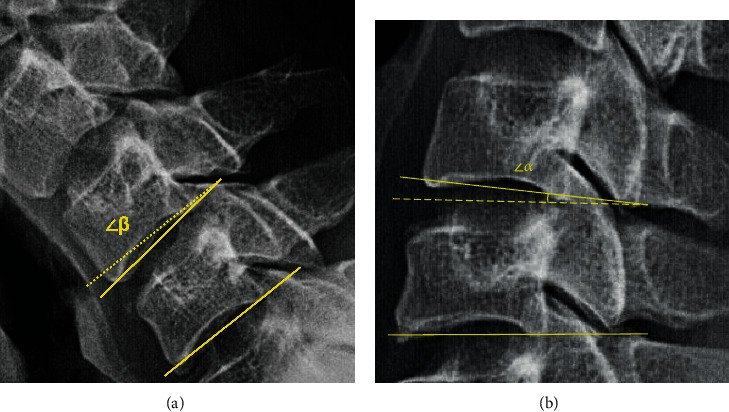
Illustration of the ROM measurement (ROM = ∠*α* + ∠*β*). The lateral view of X-ray in the (a) flexion and (b) extension posture.

**Figure 5:**
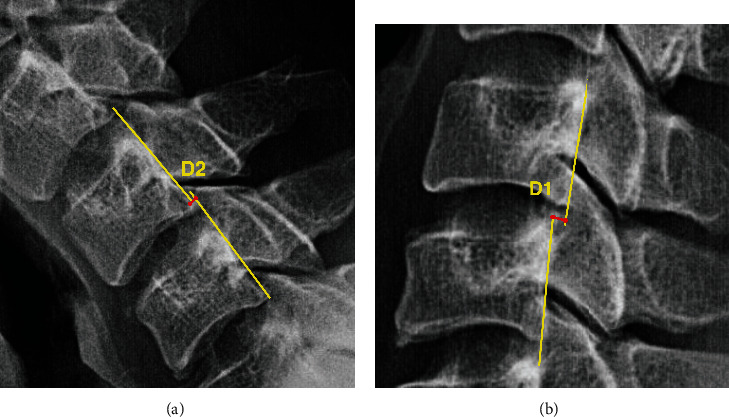
Illustration of the distance of slippage (D = D1 + D2). The lateral view of X-ray in the (a) flexion and (b) extension posture.

1. To evaluate clinical efficacy evaluation, the NDI, VAS for arm/neck pain, and modified MacNab criteria were used
2. Regarding the imaging evaluation, the range of motion (ROM) (Figure 4) and the slippage distance (D) (Figure 5) were measured to evaluate the stability of the surgical segment according to the cervical flexion-extension X-ray. The ROM is defined as the sum of the angles of adjacent intervertebral motion in flexion and extension. The slippage distance is defined as the displacement between adjacent vertebrae in flexion and extension radiography. CT images assessed the decompression of the osseous intervertebral foramina during the first to the third day after the operation.

### 2.6. Statistical Analysis

All data were expressed as the mean ± standard deviation (SD) values and analyzed using SPSS 25.0. The Wilcoxon signed-rank test was used to compare the data recorded before and after surgery. *P* values < 0.05 were considered statistically significant.

## 3. Results

All surgeries were completed without conversion to open surgery or severe neurovascular complications. The mean surgical duration was 104.2 ± 11.8 minutes (range: 85-131 minutes). Arm pain occurred in one patient after surgery and was significantly improved three months after the operation. Two patients had sustained fingers numbness until six months after surgery, and the sensory disturbance was mainly relieved at the last follow-up. The neck VAS and upper limb VAS scores significantly decreased postoperatively (*P* < 0.001, *P* < 0.001). The NDI significantly decreased from 32.7 ± 6.7 preoperatively to 8.6 ± 4.2 at the last follow-up (*P* < 0.001). The modified MacNab grading criteria were excellent in 19 cases, good in 3 cases, and fair in 2 cases on the third postoperative day. The satisfaction rate was 91.7% (22/24). At the last follow-up, 23 cases were considered excellent, and only 1 case was considered good. The satisfactory rate was 100% (24/24) (Table 2). The ROM of the surgical segment and the slippage distance in flexion-extension did not significantly change from preoperatively to the last follow-up (*P* = 0.968, *P* = 0.394) (Table 3).

## 4. Discussion

In 1858, Von Luschka first described the Luschka joint, a unique anatomical structure of the cervical spine, and maintained the mobility and stability of the cervical spine motion segment. Additionally, it protected the intervertebral foramina and prevented intervertebral disc herniation from the lateral side [20, 21]. In the process of cervical degeneration, osteophytes with the Luschka joint hyperplasia could lead to cervical intervertebral foraminal stenosis, which was a common cause of neck and upper extremity pain. For cervical intervertebral foraminal stenosis treatment, anterior and posterior surgical approaches had been reported and achieved satisfactory clinical results. However, the optimal treatment of cervical osseous foraminal stenosis (COFS), primarily caused by uncinate process hyperplasia, was still controversial [4–7, 9, 13–15].

Anterior cervical discectomy and fusion (ACDF) is the gold standard for managing cervical neck pain and radiculopathy. Patients with clinical evidence of radiculopathy and imaging findings consistent with anterior nerve root impingement may benefit from ACDF. As the exiting cervical nerve roots are closely related to the posterior aspect of the uncovertebral joint, indirect foraminal decompression through distraction may play an essential role during ACDF [4]. However, it has been shown that the loss of segmental mobility after ACDF may accelerate spondylotic changes and degeneration of discs at adjacent vertebral levels due to increased stress and malnutrition from fusion [22]. Therefore, an increasing number of spine surgeons prefer to preserve the cervical spine motion segment and decompress the cervical intervertebral foramina by minimally invasive nonfusion techniques [7–9, 11–17]. Lee et al. [7] reported that the base of the uncinate process could be directly removed without destroying the intervertebral disc via microendoscopic anterior foraminotomy to treat unilateral cervical radiculopathy. Salvatore et al. [8] reported that multisegment oblique resection of the cervical vertebral body combined with anterior foraminotomy could achieve a wide range of decompression and preserve the physiological activity and stability of the cervical spine. Cervical anterior nonfusion decompression surgery could achieve direct nerve decompression with satisfactory clinical outcomes and retain the function of the intervertebral disc. However, the surgery is complicated and should be monitored under continuous X-ray fluoroscopy. Moreover, there is a risk of serious complications, such as injury to the carotid artery, vein, trachea, esophagus, thyroid gland, and intended ganglion.

Considering the disadvantages of ACDF and the risks of anterior surgery, the posterior approach may be a better alternative. Moreover, some studies have demonstrated that posterior foraminotomy under a microscope is an effective treatment for cervical radiculopathy [16, 17]. Recently, spinal endoscopy surgery has been advancing. The posterior full-endoscopic approach can significantly reduce iatrogenic trauma caused by surgery [11, 12, 14, 15]. Though biomechanical research showed that ACDF could widen the intervertebral foramen, the foraminotomy may be more efficient by directly decompressing the foramen with a stable cervical spine [23].

Scoville first described posterior cervical foraminotomy via partial resection of the medial margin of the facet joint to treat cervical radiculopathy in 1945 [24]. Although the posterior procedures preserved the segmental mobility, decompression of the nerve root was indirect, leaving anterior foraminal osteophytes intact. Also, osseous foraminal stenosis required more facet joint removal to widen the intervertebral foramina adequately. Instability might occur if the decompressive procedure was undertaken at multiple levels or if more than 50% of the facet joint was removed [25].

**Figure 6:**
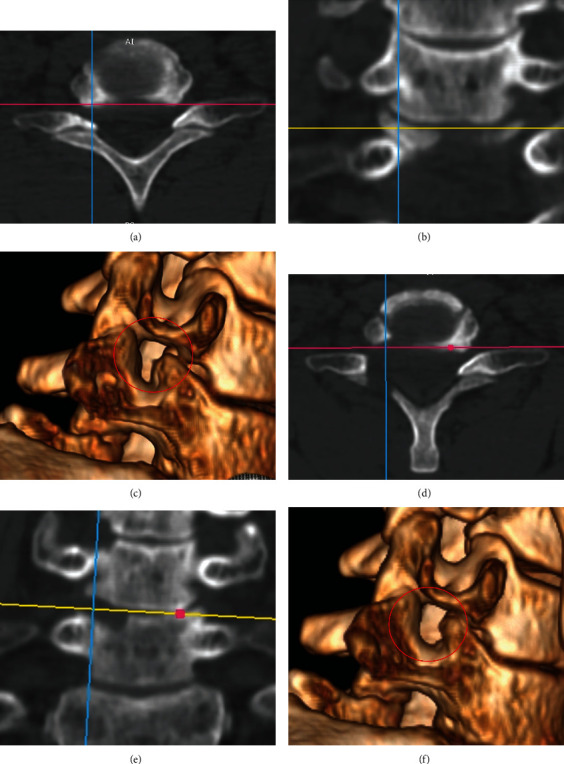
Comparison of CT images before and after decompression from the inferior of the exiting root at the C6-7 level. Preoperative images: (a) axial view, (b) coronal view, and (c) reconstruction image. Postoperative images: (d) axial view, (e) coronal view, and (f) reconstruction image.

**Figure 7:**
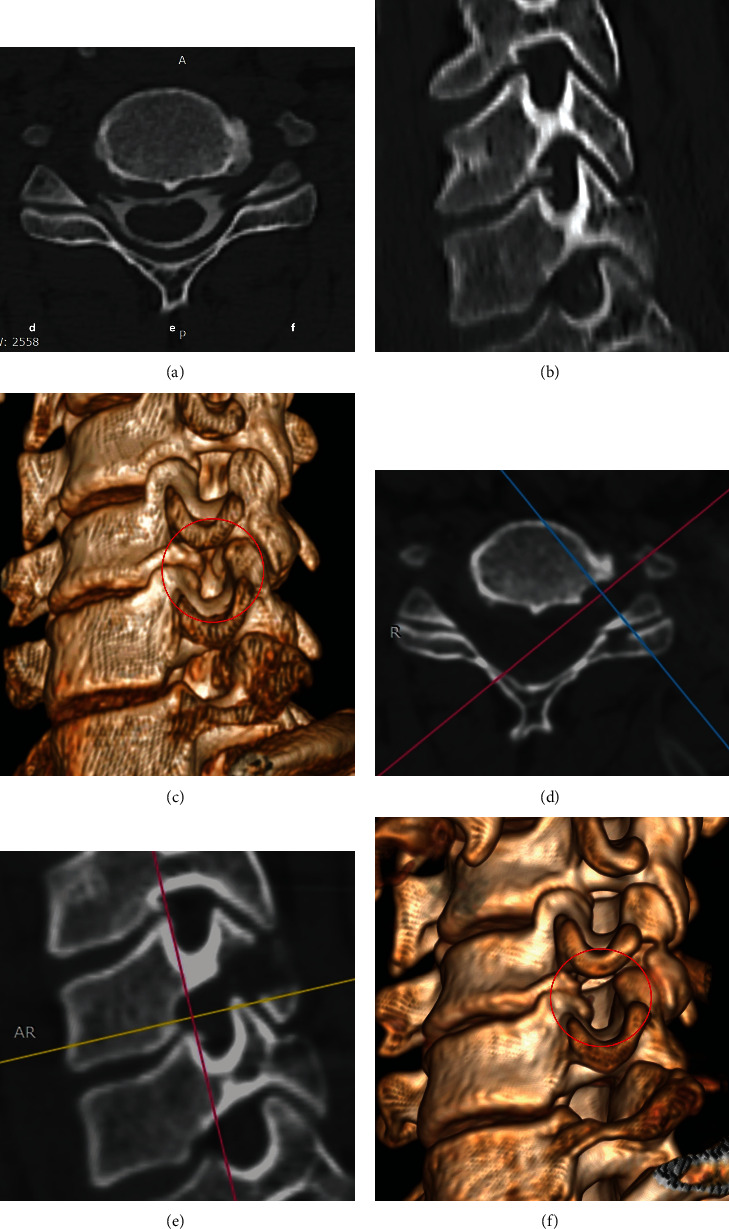
Comparison of CT images before and after decompression from the superior of the exiting root at the C5-6 level. Preoperative images: (a) axial view, (b) sagittal view, and (c) reconstruction image. Postoperative images: (d) axial view, (e) sagittal view, and (f) reconstruction image.

Although some researchers had improved the posterior foraminotomy techniques [19], which could significantly reduce the resection of the posterior facet joints, there was still no literature describing how to remove the anterior osteophyte from the posterior approach. In the current study, the posterior circumferential decompression technique can decrease the risk of vertebral artery injury with adequate endoscopic visualization and intraoperative fluoroscopic guidance. The endoscopic approach can also preserve the stability of the cervical spine by minimizing the injury to the facet joint with smaller instruments. The critical point was the targeted decompression according to the position between osteophytes and nerve roots before surgery. The trajectory can be modified and determined by the osteophyte location on the preoperative CT images. We presented two cases of uncinate process hyperplasia at two different sites. In one case, stenosis in the lower part of the intervertebral foramen, decompression was performed from the inferior exiting root at the C6-7 level (Figure 6). The other one, stenosis in the upper part of the intervertebral foramen, performed decompression from the superior of the exiting root at the C5-6 level (Figure 7). As for the technique of osteophyte removal, we suggested endoscopic trephine for less likely to damage the dural sac while removing osteophytes compared with the drill. When a protective sleeve was not used, the drill was likely to entangle the surrounding soft tissue and cause dural laceration when in close contact with the dural sac. Besides, a high-speed drill produced a lot of bone dust that could blur the endoscopic visualization—handling the endoscopic drill while removing the osteophyte aside from the nerve root and dural sac is challenging. Therefore, we reached the osteophyte surface by gliding the endoscopic trephine from the axilla of the nerve root to the front and then slowly rotating to remove the osteophyte. It should be noted that squeezing the dural sac with endoscopic trephine was forbidden, and pulling the nerve root should be done under neurophysiological monitoring or local anesthesia to avoid nerve injury.

In our study, 24 patients with COFS successfully underwent posterior full-endoscopic circumferential decompression, a modified technique based on the posterior keyhole approach. However, the symptoms of one male patient with arm pain at the C5-6 segment were aggravated after surgery. Inappropriate intraoperative nerve root traction might be the reason, but total pain relief occurred three months postoperatively. Moreover, finger numbness lasted for at least six months after surgery in two female patients and was mainly relieved at the last follow-up. The long duration of nerve root compression leading to ischemia-reperfusion injury after sufficient decompression might be the primary reason for this complication. Most of the upper facet joint was resected in one patient, but no slippage or instability was found at the one-year follow-up. Moreover, we observed that bone union appeared in the bone removal area in most cases. According to the radiological outcomes in our study, posterior full-endoscopic circumferential decompression removed the osteophytes of the uncinate process and preserved most of the Luschka joint and the stability of the segment in the short-term follow-up. In short, the best indication for this technique was COFS, and the intervertebral mobility needed to be preserved. The contraindication was myelopathy or the dynamic cervical spine X-ray showed evidence of instability.

There are limitations in the current study. The case number is small, and this is an observational study. This study was based on a single surgeon's experience. Though the authors preferred PECF under local anesthesia, surgeons can also conduct PECF under general anesthesia with or without neurophysiological monitoring. The indications of PECF are limited, and the learning curve of spinal endoscopy is steep without standardized workflow. The follow-up time was short, and the long-term result is necessary to evaluate the degenerative process of the cervical spine. Further biomechanical studies are necessary to compare posterior cervical foraminotomy with circumferential decompression and traditional technique.

## 5. Conclusions

In summary, posterior full-endoscopic circumferential decompression is a feasible minimally invasive surgical technique to treat COFS. It is an innovative technique based on the traditional keyhole approach and achieved satisfactory results in the early clinical follow-up. Moreover, it preserves the stability and physiological mobility of the cervical spine.

## Figures and Tables

**Table 1:** The demographic characteristics of the patients.

Characteristics	Data
Mean age, years	51.5 (38~68)
Sex	
Male	11
Female	13
Level	
C4-5	6
C5-6	13
C6-7	5
Mean duration of symptom, months	7.4 (2~24)
Mean follow-up time, months	16.3 (12~26)

**Table 2:** Operative details and clinical outcomes.

Characteristics	Data
Operation duration (min)	104.2 ± 11.8
Surgical complications rate	12.5% (3/24)
Hospital stay (day)	7.3 ± 0.8
Modified MacNab	
3 days post-op	91.7%
Last follow-up	100%

**Table 3:** Clinical and radiological outcome scores.

Characteristics	Pre-op	Last follow-up	*P* value
VAS-neck	5.4 ± 0.8	1.3 ± 0.8	<0.05
VAS-arm	6.0 ± 0.9	1.1 ± 0.9	<0.05
NDI %^∗^	32.7 ± 6.7	8.6 ± 4.2	<0.001
ROM (°)	5.2 ± 1.8	5.1 ± 1.8	0.968
Distance of slippage(mm)	1.8 ± 0.6	1.7 ± 0.6	0.394

Pre-op: preoperation; Post-op: postoperation; VAS: visual analogue scale; NDI: neck disability index; ROM: range of motion. ^∗^ indicates significant differences between Pre-op and last follow-up (*P* < 0.05).

## Data Availability

The datasets generated and analyzed during the current study are available from the corresponding author on reasonable request.

## Supplementary Materials

Supplementary Materials The supplementary video showed the procedures of circumferential decompression by posterior endoscopic cervical foraminotomy.
